# Genomic resources for a unique, low-virulence *Babesia* taxon from China

**DOI:** 10.1186/s13071-016-1846-1

**Published:** 2016-10-27

**Authors:** Guiquan Guan, Pasi K. Korhonen, Neil D. Young, Anson V. Koehler, Tao Wang, Youquan Li, Zhijie Liu, Jianxun Luo, Hong Yin, Robin B. Gasser

**Affiliations:** 1 State Key Laboratory of Veterinary Etiological Biology, Lanzhou Veterinary Research Institute, Chinese Academy of Agricultural Sciences, Lanzhou, Gansu China; 2 Faculty of Veterinary and Agricultural Sciences, The University of Melbourne, Parkville, VIC 3010 Australia

**Keywords:** *Babesia* sp. Xinjiang, China, Sheep, Genome, Variant erythrocyte surface antigens (VESAs)

## Abstract

**Background:**

Babesiosis is a socioeconomically important tick-borne disease of animals (including humans) caused by haemoprotozoan parasites. The severity of babesiosis relates to host and parasite factors, particularly virulence/pathogenicity. Although *Babesia bovis* is a particularly pathogenic species of cattle, there are species of *Babesia* of ruminants that have limited pathogenicity. For instance, the operational taxonomic unit *Babesia* sp. Xinjiang (abbreviated here as *Bx*) of sheep from China is substantially less virulent/pathogenic than *B. bovis* is in cattle. Although the reason for this distinctiveness is presently unknown, it is possible that *Bx* has a reduced ability to adhere to cells or evade/suppress immune responses, which might relate to particular proteins, such as the variant erythrocyte surface antigens (VESAs).

**Results:**

We sequenced and annotated the 8.4 Mb nuclear draft genome of *Bx* and compared it with those of *B. bovis* and *B. bigemina* by synteny analysis; we also investigated the genetic relationship of *Bx* with selected *Babesia* species and related apicomplexans for which genomic datasets are available, and explored the VESA complement in *Bx*.

**Conclusions:**

The availability of the *Bx* genome now provides unique opportunities to elucidate aspects of the molecular biology, biochemistry and physiology of *Bx*, and to explore the reason(s) for its limited virulence and/or apparent ability to evade immune attack by the host animal. Moreover, the present genomic resource and an *in vitro* culture system for *Bx* raises the prospect of establishing a functional genomic platform to explore essential genes as new intervention targets against babesiosis.

**Electronic supplementary material:**

The online version of this article (doi:10.1186/s13071-016-1846-1) contains supplementary material, which is available to authorized users.

## Background

Babesiosis is a globally important tick-borne, parasitic disease of animals, including humans, caused by haemoprotozoans of the genus *Babesia* (phylum Apicomplexa). This disease has a major, adverse economic impact on the health and productivity of livestock animals, particularly ruminants, as a consequence death, reduced meat and milk production, increased sterility and abortion rates and/or the cost of treatment and prevention [1], and is an ongoing problem particularly in tropical and subtropical regions of Australia, Africa and the Americas. Most economic impact appears to be linked to bovine babesiosis, caused by *Babesia bovis* and *B. bigemina*, but the socioeconomic importance of babesiosis in small ruminants is also likely to be considerable in some countries [2, 3].

*Babesia* spp. are transmitted to their mammalian hosts by particular ixodid tick species. The tick injects sporozoites into the blood stream upon feeding; these ‘zoites directly invade the erythrocyte and undergo asexual replication (binary fission) to produce many merozoites that are released into the circulation following erythrocyte rupture and then reinvade erythrocytes, and the cycle continues. This rapid, perpetual cycle of replication (merogony) and associated erythrocyte invasion and destruction usually lead to intravascular haemolysis, anaemia, haemoglobinuria and/or jaundice. The severity of disease usually relates to host and parasite factors, but often the virulence/pathogenicity is of considerable importance. For example, *B. bovis* is particularly pathogenic in *Bos taurus* and can dramatically modify the structure and functionality of infected erythrocytes [4, 5]; this alteration can be accompanied by an accumulation of affected erythrocytes in the capillaries of organs, including the brain and lungs, leading to severe cerebral disease, respiratory insufficiency and/or multi-organ failure. Interestingly, in contrast to *B. bovis*, there are species of *Babesia* of ruminants that have limited pathogenicity. For example, the operational taxonomic unit *Babesia* sp. Xinjiang (abbreviated as *Bx*) of sheep from central and northwestern regions of China, which is transmitted by *Hyalomma anatolicum anatolicum*, has been reported to have limited virulence/pathogenicity in sheep (*Ovis aries*) [6, 7]. Although the reason/s for this observation is/are not yet known, it is possible that *Bx* has a reduced ability to adhere to cells or evade/suppress immune responses, which might relate to particular protein groups, including variant erythrocyte surface antigens (VESAs) and/or small open reading frame (SmORF) proteins [8].

The availability of an effective and continuous *in vitro*-culture system for *Bx* [9] provides unique opportunities for detailed investigations of antigenic variation, virulence factors, the parasites’ biology and molecular biology *via*, for instance, functional genomics [5], with a future prospect of discovering new intervention methods against babesiosis more generally. To provide a foundation for such research areas, in the present study, we (i) sequenced the nuclear genome of *Bx* and compared its first draft genome with those of *B. bovis* and *B. bigemina* by synteny analysis; (ii) studied the genetic relationship of *Bx* with other *Babesia* species and related apicomplexans for which genomic datasets are available; and (iii) explored the complement of *ves* genes and their predicted proteins in *Bx*.

## Methods

### Sequencing and preparation of data

Merozoites of *Babesia* sp. Xinjiang [7] were maintained in sheep erythrocytes in a continuous *in vitro* culture and amplified in a parasite-free, splenectomised sheep [9]. Merozoites were purified from blood as described [10], and genomic DNA was isolated using the Gentra Puregene kit (Qiagen, Hilden, Germany) and total RNA employing TriPure (Sigma-Aldrich, St Louis, MI, USA), according to the manufacturers’ protocols. The nucleic acids were quantitated using a fluorometer (Qubit*,* Invitrogen, Carlsbad, CA, USA), and their quality was verified using a BioAnalyzer (2100, Agilent). One paired-end (500 bp insert size) and two mate-pair (2 kb and 5 kb) genomic DNA libraries were sequenced using Illumina technology (HiSeq; 2 × 100 reads for paired-end libraries, and 2 × 49 reads for mate-pair libraries), and RNA-sequencing (RNA-seq) was conducted using an established protocol (Illumina). Genomic and RNA-seq reads were quality-filtered using the program Trimmomatic v.0.36 [11], and RNA-seq reads were processed further using Khmer v.2.0 [12].

### Prediction of repetitive elements

First, genomic repeats were modelled using the program RepeatModeler [13], and repeat predictions merged using the programs RECON [14], RepeatScout [15] and Tandem Repeat Finder (TRF) [16]. Second, long terminal repeats (LTRs) were predicted using the program LTR_Finder [17]. Third, simple repeats and transposons were predicted using RepeatMasker v.4.0.5 [18], with transposons being predicted using data from Repbase v.17.02 [19]. Fourth, all repeats were combined using RepeatMasker v4.0.5.

### Genomic assembly and gene prediction

Short-read data were assembled using the program SPAdes v3.5.0 [20] and scaffolded using the program SSPACE v3.0 [21]. Genes were predicted with MAKER2 [22] using the msoftware suite containing the *ab initio*-gene prediction programs AUGUSTUS [23], GeneMark-ES [24] and SNAP [25]. Genome-guided gene predictions using RNA-seq read data were conducted using TopHat2 v2.1.0 [26] and Cufflinks2 v2.2.1 [27]. RNA data were assembled using both *de novo-* and genome-guided approaches using the Trinity platform [28]. The resultant transcriptome, together with a set of proteomes from NCBI protein database [29] for *B. bovis*, *B. microti*, *Cryptosporidium hominis*, *C. muris*, *C. parvum*, *Neospora caninum*, *Plasmodium falciparum*, *Theileria annulata*, *Th. parva*, *Toxoplasma gondii* and *Tetrahymena thermophila*, was used as ‘evidence data’ for gene prediction. EVidenceModeler (EVM) software [30] was utilised to combine gene predictions as well as protein sequence and transcript alignments into weighted consensus gene structures. In short, the transcriptomic data set was mapped to the genome using the pipeline PASA2 [30]; the resultant gene predictions, transcriptome and proteome mappings from the MAKER2 prediction were then integrated using EVM. The resultant protein-coding gene set was then consolidated using the following approach: (i) genes containing repeats that overlapped by ≥ 80 %, had ≤ 20 % transcript support and whose codon usage was consistent with a coding region, as established using program TransDecoder (within the Trinity), were removed; and (ii) genes containing repeats that overlapped by ≥ 80 % or had ≤ 20 % transcript support and whose codon usage was *not* consistent with a coding region were removed. Finally, the tRNA genes were predicted using the program tRNAscan-SE [31]. For the predicted genes, the genome completeness was estimated using the program Benchmarking Universal Single Copy Orthologs (BUSCO) [32].

### Genome annotation

Protein-coding genes were annotated using the programs InterPro [33] and BLAST+ [34, 35]. BLAST+ was applied to the proteome of *B. bovis* [36], and to the databases UniProtKB/SwissProt [37], KEGG [38] and NCBI protein nr [39]. Signal peptides were predicted using SignalP [40] and transmembrane protein regions employing TMHMM [41]. A custom script was created to convert the assembly, the predicted genes and the gene annotations into Abstract Syntax Notation One (ASN.1) for NCBI submission. The program Genome Annotation Generator (GAG) v1.0 [42] was used in this custom script.

### Phylogenetic analysis

First, single copy orthologous (SCO) protein-coding genes shared by the proteomes of 16 species (*Bx*, *B. bovis*, *B. bigemina*; *C. parvum*, *C. hominis*; *Eimeria tenella*; *P. chabaudi, P. falciparum*, *P. knowlesi, P. vivax*; *Th. annulata, Th. equi, Th. orientalis, Th. parva*; *To. gondii*; and *Te. thermophila*) were identified using the program OrthoMCL [43, 44]. The 16 amino acid sequences representing individual SCOs were aligned using the program MAFFT v7.271 [45], and the SCOs with a minimum gap-free alignment length of 20 amino acids and with at least one phylogenetically informative site were selected. The final subset of SCOs common to all 16 species were then concatenated and subjected to phylogenetic analyses using the methods Bayesian inference (BI) in MrBayes v.3.2.2 [46, 47] and Maximum Likelihood (ML) in RAxML v.8.0.24 [48]; *Te. thermophila* was used as an outgroup. For BI, following the model selection using the program ProtTest 3.4 [49], the prior evolution model for amino acids was set to WAG [50], and the likelihood model was set to invgamma [51, 52]; from 200,000 Markov Chain Monte Carlo (MCMC) [53–55] iterations, the first 50,000 were discarded as non-converged burn-in, and nodal support values were given as posterior probabilities. For ML, the JTT [56], evolution model was used and the concatenated alignment blocks were bootstrapped 100 times to infer nodal support values. Phylogenetic trees were drawn using FigTree v1.4 (http://tree.bio.ed.ac.uk/software/figtree/).

### Synteny

Synteny among *Bx*, *B. bovis* and *B. bigemina* was established using a custom script. The scaffold-pairs containing SCOs shared between the two species were converted into a bipartite graph and processed in a one-sided crossing minimization algorithm [57] employing the program DSDP5 (a software for semi-definite programming) [58].

### Variant erythrocyte surface (*ves*) antigen genes

These genes were first predicted using the program BLASTp (E-value 10^-8^) in the annotation of the draft genome for *Bx* and then using the HMM models for *B. bovis*, *B. bigemina* and *B. divergens* [8]. The predicted genes were then aligned using the program MAFFT, and shared amino acid patterns of encoded proteins identified manually and using the program PRATT v2.1 [59]. The program HMMER v3.1.2 [60] was used to search for the genes encoding proteins with a VESA1_N domain for variants a and b, listed in the Pfam database [61]. The matching domain sequences were extracted from protein sequences using a custom script and aligned using MAFFT. A *Bx*-specific VESA1_N HMM model was created using the program HMMER. This model was used to predict the VESA1_N domain in predicted proteins of *Bx*. The *ves* genes encoding proteins with this domain as well as shared patterns were drawn using a custom script.

## Results and discussion

The draft nuclear genome of *Bx* is 8.4 Mb in size (Table 1). We detected 195 of 429 core essential genes by BUSCO, suggesting a near complete genome. The *Bx* genome is similar in size with the congeners *B. bovis* (8.2 Mb), but smaller than *B. bigemina* (13.8 Mb) and larger than *B. microti* (6.5 Mb) [8, 36, 62]. We estimated the repeat content of this draft genome at 4.3 %, equating to 365.6 kb, of which interspersed repeats comprised 145 LINEs, 5 DNA and 431 unclassified elements.

**Table 1:** Features of the draft genome of *Babesia* sp. Xinjiang (*Bx*) with those of *B. bovis*, *B. bigemina* and *B. microti*

Features	*Babesia* sp. Xinjiang (*Bx*)	*Babesia bovis*	*Babesia bigemina*	*Babesia microti*
Genome size (Mb)	8.4	8.2	13.8	6.5
Number of scaffolds or chromosomes	215	4	6	3
N50 for scaffolds (kb)	533.30	–	3520	–
N90 for scaffolds (kb)	96.98	–	–	–
Genome GC content (%)	43.9	41.5	50.6	36.0
Repetitive sequences (%)	4.3	–	–	–
Exonic proportion/incl. introns (%)	63/71	70/73	–/63	73/81
Number of nuclear protein-coding genes	3066	3706	4457	3513
Gene density (bp per gene)		2194	2306	1816
Mean gene length including introns (bp)	1958	1609	1531	1471
Mean CDS length (bp)	1721	1503	–	1327
Mean exon number per gene	3.3	2.8	–	3.3
Mean exon length (bp)	530	547	–	397
Mean intron length (bp)	106	60	–	61
Coding GC content (%)	45.4	44.0	51.7	39.0
Number of predicted tRNAs	41	70	–	44
BUSCO completeness (%/count)	45/195	48/204	49/210	49/212

**Fig. 1:**
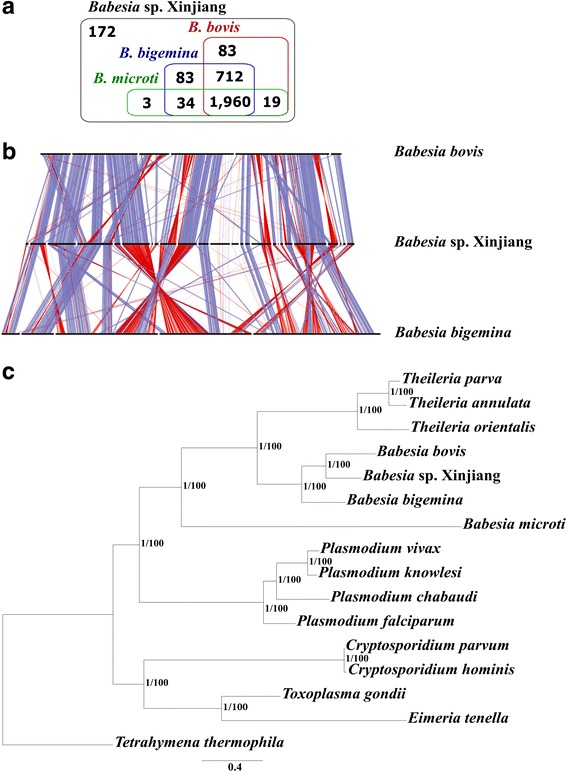
**a** VENN diagram for the *Babesia* sp. Xinjiang (*Bx*) genes orthologous to those of *B. bovis*, *B. bigemina* and *B. microti*. Altogether, 1960 genes in *Bx* are shared with three other *Babesia* species, and 172 genes are unique to *Bx*. **b** Single copy orthologous (SCO) genes (*n* = 2136) among the scaffolds in the draft genomes of *B. bovis*, *Bx* and *B. bigemina* in the forward (*blue*) or reverse orientation (*red*). **c** Phylogenetic tree constructed from sequence data for SCOs (*n* = 326) shared among all apicomplexans for which proteomic data were available. The nodal support values were all 1.00 (posterior probability; pp) and 100 % (bootstrap), indicated as ‘1/100’

We annotated and then compared the gene set of *Bx* with those of *B. bovis, B. bigemina* and *B. microti* as well as other selected apicomplexans. In the draft genome of *Bx*, we identified 3066 protein-coding genes, 754 of which were supported by transcriptomic data for merozoites, with a mean total length of 1.96 kb, mean exon length of 530 bp and a mean of 3.3 exons per gene (see Table 1). Approximately 96.8 % (*n* = 2969) of the predicted *Bx* genes (Fig. 1) have an homolog (BLASTp cut-off: 10^-8^) in *B. bovis* (2874; 92.7 %), *B. bigemina* (2907; 94.8 %) or *B. microti* (2227; 72.6 %) [8, 36, 62]. A total of 1960 *Bx* genes are orthologous (OrthoMCL BLASTp cut-off 10^-8^) among all four taxa of *Babesia*, and 2894 were shared by at least one other taxon (Fig. 1). Conversely, 172 (5.6 %) genes are unique to *Bx* (Fig. 1). Of the entire *Bx* gene set, 984 genes had an ortholog (≤10^-8^) linked to 246 known biological pathways (see Additional file 1). Comparison of universal SCOs among these four *Babesia* taxa (cf. Table 1; *n* = 195 for *Bx* and *n* = 204–212 for others) indicates that the majority of *Bx* genes are represented in the present genomic assembly for *Bx*.

As the specific status of *Bx* has not yet been resolved, we were keen to assess its evolutionary relationship with known *Babesia* species and other apicomplexan haematoprotozoa (*Theileria* and *Plasmodium*) for which published genomes were available. Based on two independent analyses of sequence data for 326 shared SCOs, we showed that *Bx* from sheep was more closely related to *B. bovis* than to *B. bigemina* from cattle, a finding supported by a genome-wide syntenic comparison among the three taxa (Fig. 1). Although the number of genome sequences publicly available for *Babesia* is presently limited, this finding is interesting, given the discrepancy in pathogenicity between *Bx* and *B. bovis.* Of note was also the result that species of *Babesia* and of *Theileria* each grouped together (and grouped with one another) to the exclusion of *B. microti*, suggesting that the latter species does not belong to either the genus *Babesia* or *Theileria*. This finding is supported by previous evidence from other phylogenetic analyses using data representing a small number of genetic markers (e.g. [63–65]) and 316 genes [62]. The present results show that *Babesia* is a paraphyletic group, indicating that the taxonomy of members of this genus needs to be revised; they also show that *B. microti* has diverged early during piroplasm evolution. Overall, these findings also suggest that *B. microti* represents a new genus that is distinct from both *Babesia* and *Theileria*, in accord with a previous proposal [62].

**Fig. 2:**
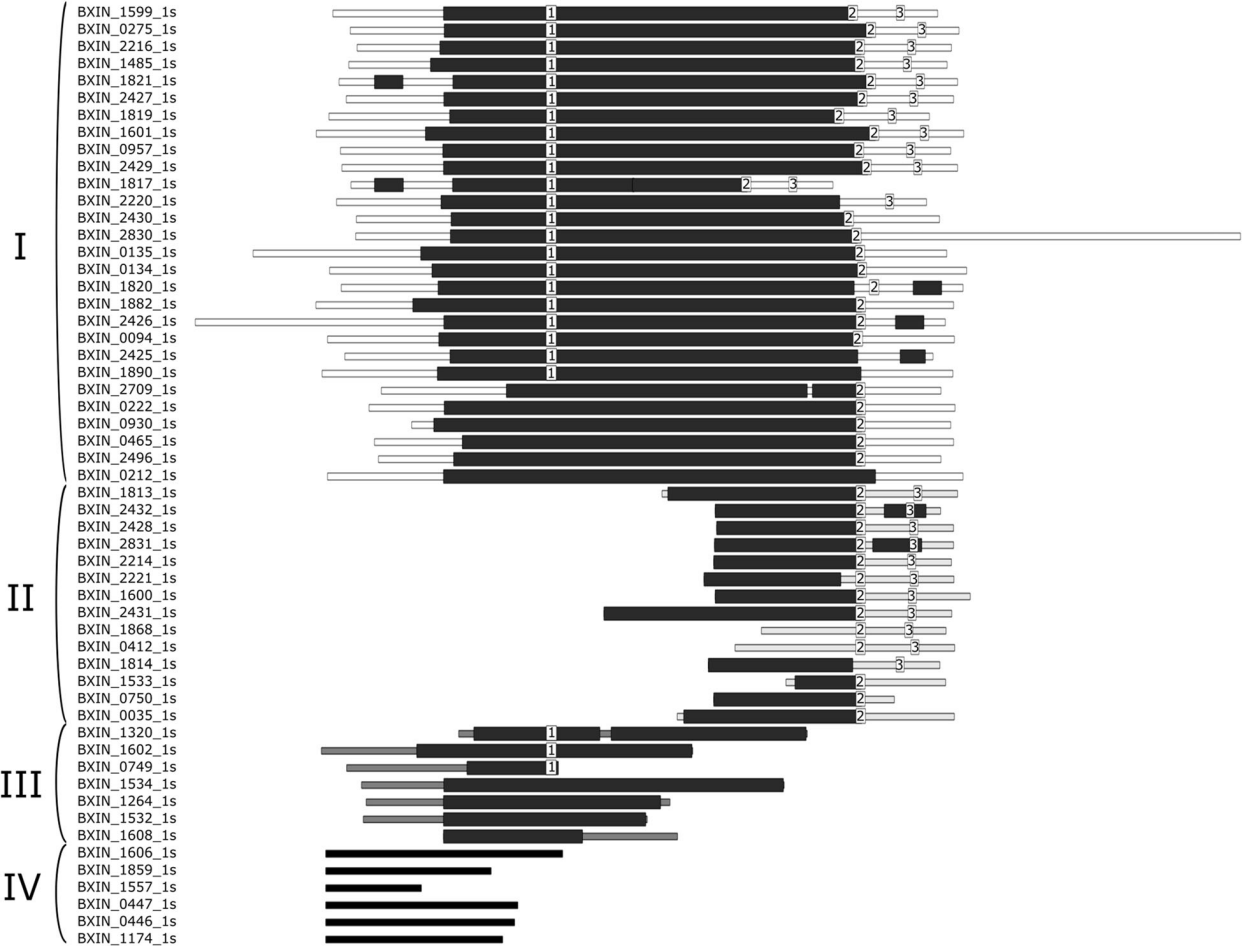
Summary of the 59 variant erythrocyte surface antigens (VESAs) encoded by *ves* genes of *Babesia* sp. Xinjiang. The Pfam domain VESA1_N (*dark grey* broad rectangles) and three amino acid patterns [ST]IREMLYWLMXLP[YS] (box 1), CXCXXXVXCXXXL (box 2) and PF[LF][LFY]YLLTFWL (box 3) are indicated. These VESAs were assigned to four distinct groups (I to IV)

Although *Bx* appears to be closely related to *B. bovis* (cf. Fig. 1), there is a distinct difference between these two species in their pathogenicity in their respective host animals. As indicated, on one hand, *Bx* is virtually non-pathogenic in susceptible sheep (and not infective to calves or goats) [9], whereas *B. bovis* is highly pathogenic in the naïve bovine host [5]. This evidence appears to indicate a considerable distinctiveness in the *Bx*’s ability to evade or suppress host immune responses. Given that variant erythrocyte surface antigens (VESAs) encoded by *ves* genes [8, 36] have been implicated in immune evasion/modulation, the pathogenesis and/or the persistence of infections in the host, we focused our attention here on investigating the nature and extent of genes encoding these molecules in *Bx*. Initially, the protein sequences homologous to those encoded by the *ves* genes of *B. bovis*, *B. bigemina* and *B. divergens* were identified. From the results, it became evident that VESAs in *Bx* were substantially distinct both in number and sequence from those of *B. bovis*, *B. bigemina* and *B. divergens*, such that they could not be classified in the same way as for their congeners. Therefore, we defined three distinct patterns that typify the 59 VESAs encoded in *Bx*, and used these patterns as well as the VESA1_N domain to classify four distinct groups of VESAs (I-VI; Fig. 2): Specifically, group I proteins (*n* = 28) share the VESA1_N domain and have one or more additional patterns; group II proteins (*n* = 14) share a short domain and/or a pattern near the C-terminus (long proteins); group III proteins (*n* = 7) share a short domain and can have the pattern near the N-terminus (long proteins); group VI proteins (*n* = 6) have no domain or pattern. Based on these results, it is evident that VESAs and their genes are highly labile or plastic in terms of genome repertoire and sequences, suggesting that the substantial divergence observed relates to frequent transposition to new genomic positions over time. Previous phylogenetic analyses of *ves* gene repertoires from various strains of *B. bovis*, *B. bigemina* and *B. divergens* from distinct geographical localities did not indicate strain-associated gene family expansions [8]; gene transposition appeared to be more frequent than evolution through amino acid substitution or gene duplication [8]. Moreover, most *ves* genes in *Bx*, *B. bovis* and *B. bigemina* are not orthologous, even though they are relatively conserved in their position in the genome. Together with previous results [8], the present findings seem to support the proposal for a key role of recombination in *Babesia*, and that genomic architecture enables recombination to promote antigenic diversity and/or switching [66]. Consistent with *var* genes of *Plasmodium* [67], rapid gene turnover, recombination and structural change appear to be responsible for *ves* gene diversity and complexity within and among *Babesia* species and their ability to induce disease and/or modulate or suppress host immune responses.

## Conclusions

Although there have been some improvements in our understanding of the molecular biology of *Babesia*, progress has been relatively slow, as only a relatively small number of researchers around the world are investigating these apicomplexans. The genome of *Bx* provides a new and exciting resource for many future studies. Progress could proceed along many different lines.

One might be to complete the genome and sequence various species and strains of *Babesia* from small ruminants (sheep and goats) to chromosome-scale contiguity. Such an effort would resolve regions of tandem multigene families, which are often absent from assemblies of short read (Illumina) data sets [68], but that are central to understanding species- and/or strain-specific traits [69, 70]. Another aspect could be to undertake detailed comparative analyses of the genome and transcriptome of *Bx* with other apicomplexans. Genomic comparisons could identify genes that are undergoing positive selection or gene family expansions or contractions in particular *Babesia* species, and may, therefore, provide insights into the evolution of gene families and their (possible) roles in virulence, pathogenicity and parasitism. Another avenue of investigation might be to explore the transcriptome of *Bx* in more detail and which genes or gene families are involved in parasitism. It would also be interesting to characterize stage-specific transcripts as well as ncRNAs to establish their contributions to a parasitic mode of existence. Moreover, the transcriptome of *Bx* could be used to model the parasite’s metabolism (cf. [71]), which could be of considerable value if extended to other *Babesia* species.

Clearly, there are many fundamental areas to tackle, to elucidate the biology of *Bx* and its relatives. In our opinion, a focus on molecular aspects of virulence, pathogenesis of disease, immune evasion or suppression as well as gene function would be particularly interesting, and could guide the discovery of new intervention strategies. With the availability of *in vitro* cultures for the maintenance and propagation of selected taxa, such as *Bx* [9] and *B. bovis* [8], there is now excellent potential to accelerate research of *Bx*, and gain a deep understanding of its fundamental molecular biology and its differences from *B. bovis*. The ability to stably and transiently transform *B. bovis*, and genetically manipulate its genome [72–76] raises some prospect for developing a functional genomic platform for *Bx*. Having such a platform in place would enable systems biological investigations using complementary genomic, transcriptomic and proteomic tools. It might also underpin applied research focused on developing new interventions, such as anti-*Babesia* drugs or vaccines.

## Additional file

Additional file 1: KEGG pathways for *Babesia* sp. Xinjiang. (XLSX 38 kb)

## Abbreviations

BUSCO: Benchmarking Universal Single Copy Orthologs
*Bx*: *Babesia* sp. Xinjiang
KEGG: Kyoto Encyclopedia of Genes and Genomes
RNA-seq: RNA-sequencing
SCO: Single Copy Ortholog
*ves*: Variant erythrocyte surface antigen gene
VESA: Variant Erythrocyte Surface Antigens (VESAs)
